# Image-based multiphysics modelling of random metallic meshes

**DOI:** 10.1038/s41598-026-62432-3

**Published:** 2026-07-28

**Authors:** Dimitrios Charaklias, Dayuan Qiang, Robert Dorey, Iman Mohagheghian

**Affiliations:** University of Surrey, Guildford, Surrey, GU2 7XH UK

**Keywords:** Engineering, Materials science, Mathematics and computing, Physics

## Abstract

Bio-inspired conductive networks are increasingly exploited in transparent heaters and smart multifunctional structures due to their inherent scalability, robustness, and efficient transport properties, yet their intrinsic disorder poses major challenges for quantitative modelling. Here we introduce a computationally efficient, image-driven framework for the automated digitisation, reconstruction, and multiphysics analysis of random conductive meshes. High-resolution microscopy images are converted into graph-based network representations via skeletonization, branch tracking, and connectivity refinement, preserving local geometry while drastically reducing degrees of freedom. An analytical model is developed to predict sheet resistance directly from extracted network metrics, enabling rapid, non-destructive electrical characterisation. The digitised networks are further used to perform coupled electro-thermal and electro-thermo-mechanical finite-element simulations. Experimental validation using silver mesh heaters embedded in polymer laminates shows excellent agreement with predicted electrical resistance, temperature evolution, and stiffness modulation under electrical loading. The framework reduces analysis time and enables systematic assessment of structural features, such as dangling branches, on device performance. This approach provides a scalable route for predictive design and optimisation of fractal-based multifunctional electronic materials.

**Supplementary Information:**

The online version contains supplementary material available at https://doi.org/10.1038/s41598-026-62432-3.

## Introduction

Hierarchical interconnected architectures are widely observed in biological systems, particularly in the vein architectures of insect wings, leaf venation networks, and plant root systems. These branching structures provide multiple functional advantages, including mechanical reinforcement, efficient transport, and enhanced resilience. For example, insect wing veins offer lightweight structural support and improved damage tolerance^1^, leaf venation improves water and nutrient delivery^2^, and root systems maximize soil exploration while minimizing metabolic cost^3^. Together, these examples demonstrate how hierarchical network architectures have evolved to achieve an effective balance between efficiency, robustness, and adaptability.

Hierarchical network architectures differ fundamentally from conventional layouts such as periodic grids and linear arrays. Whereas traditional designs prioritize geometric regularity and uniformity, bio-inspired networks exhibit controlled irregularity, multiscale connectivity, and branching characteristics that resemble natural growth processes. These characteristics can provide functional advantages, including increased surface area, shorter transport pathways, enhanced connectivity, and improved tolerance to localized damage^4^. In engineered systems, such attributes have been associated with improved thermal transport, electrical conductivity, and mechanical reliability^5^. Notably, many conductive metallic mesh structures used in flexible electronics, transparent conductors, and electro-thermal devices possess partially stochastic or pseudo-random geometries that share key topological features with natural branching networks, including hierarchical connectivity and multiple transport pathways.

In applied physics and materials science, bio-inspired conductive architectures are increasingly explored for optoelectronic and energy applications. In transparent heaters, conductive networks maintain optical transparency while providing high electrical connectivity, uniform current distribution, and reduced thermal hotspots^6^. In photovoltaics, hierarchical electrodes and light-management structures enhance absorption, improve charge collection, and reduce recombination losses through increased interfacial area and optimised transport pathways^7,8^. These advantages arise from the intrinsic geometry and connectivity of hierarchical networks and highlight their potential for scalable devices. Beyond uniform heating, hierarchical conductive pathways can also be used to modulate mechanical stiffness via Joule heating^9,10^. This capability is valuable in flexible and wearable systems requiring adaptive compliance and conformability. Distributed pathways and structural redundancy further enable localized control of heat delivery, allowing spatial tuning of stiffness while preserving optical and electrical functionality^11^.

The growing interest in bio-inspired design has driven the development of computational tools for quantitative analysis of branching architectures. Image-based network reconstruction frameworks, such as NEFI, enable automated identification of nodes and branches through image preprocessing, skeletonization, and graph extraction algorithms, allowing calculation of metrics such as branch lengths, node degrees, and network connectivity^12^. Similar image-analysis approaches have been employed for the characterization of biological transport systems, vascular structures, crack networks, and conductive metallic meshes^13^.

Beyond image-based reconstruction, homogenization and multiscale modelling approaches have also been widely used to estimate the effective electro-thermal behaviour of conductive networks while reducing computational cost by approximating complex geometries as equivalent continuum materials with effective properties^14,15^. Although these methods provide efficient large-scale modelling capabilities, they often neglect localized connectivity variations, junction effects, and spatial heterogeneity that strongly influence current distribution, Joule heating, and thermo-mechanical response in irregular conductive meshes.

Direct Finite Element Modelling (FEM) of detailed network geometries provides an alternative approach capable of capturing such local effects with greater fidelity, particularly in coupled electro-thermal and electro-thermo-mechanical analyses. Implementing this approach requires digital reconstruction of the network geometry, typically in a CAD-compatible format, followed by discretization for numerical simulation. However, directly converting high-resolution network images into finite element models can result in excessive meshing complexity, long simulation times, and substantial memory requirements. As network resolution and connectivity increase, these computational demands can limit scalability, design optimization, and rapid design iteration, highlighting the need for computational efficiency. Consequently, there is a need for automated modelling frameworks that preserve network topology while minimizing unnecessary computational overhead.

To address these challenges, we implemented a computationally efficient framework involving automated network extraction, including image preprocessing, skeletonization, node detection, and graph generation, followed by digital reconstruction and coupled electro-thermal and electro-thermo-mechanical simulation using FEM. An analytical model for estimating sheet resistance was also developed to support rapid electrical characterization of the extracted networks. A key advantage of the present image-driven framework is its ability to preserve the original network topology and geometric features while significantly reducing computational cost. This enables detailed investigation of localized current concentrations, thermal hotspots, and topology-sensitive effects, such as the influence of dangling branches and local pathway density. While the metallic meshes investigated here exhibit relatively uniform global temperature fields, retaining local transport heterogeneity remains important for reliability assessment, design optimisation, and extending the framework to architectures where localized electro-thermal effects may directly influence system-level behaviour.

## Methodology

### Network extraction

**Fig. 1:**
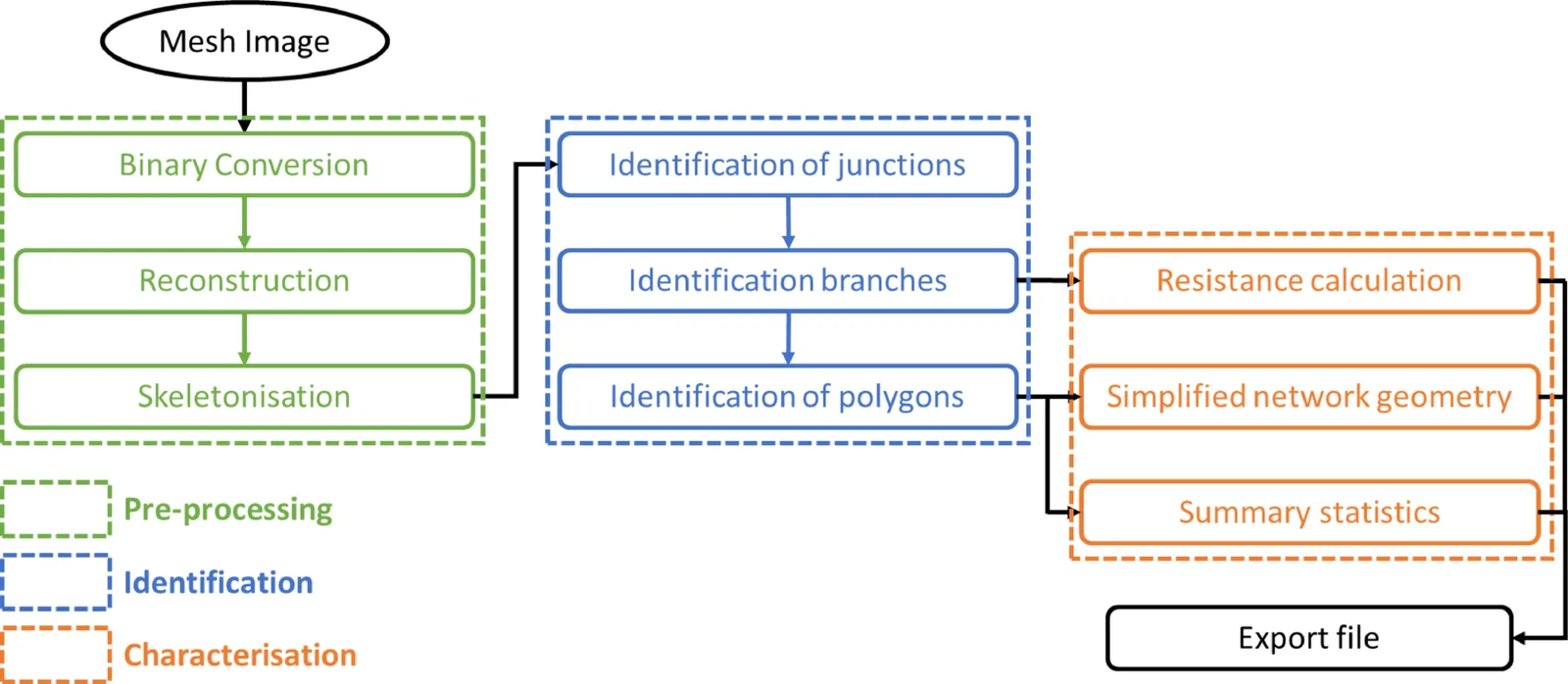
Flowchart of the digitisation steps performed within the algorithm.

Direct digitization of random metallic meshes produces models with high degrees of freedom, leading to substantial computational cost. Accurate characterisation therefore requires pre-processing to suppress noise and artefacts that hinder branch detection and width extraction. This work introduces an image-processing algorithm that simplifies network geometry while preserving physical accuracy. The overall workflow, including pre-processing, identification, and characterization, is shown in Fig. 1.

Images of manufactured crackle patterns were acquired using a Keyence 7000 Series digital microscope, manually optimised for focus and illumination to minimize distortion and ensure clear boundary definition. High-resolution tiles are automatically stitched into a composite network image, stored as a.tif raster. The microscope output is an $\:M\times\:N\times\:3\:$RGB matrix, where * M* and * N* denote image dimensions and the third axis corresponds to red (*R*), green (*G*), and blue (*B*) channels. To reduce data size, the image is converted to greyscale ($\:M\times\:N\times\:1$) using:

1 $$\:Y=0.2125\times\:R+0.7154\times\:G+0.0721\times\:B$$

**Fig. 2:**
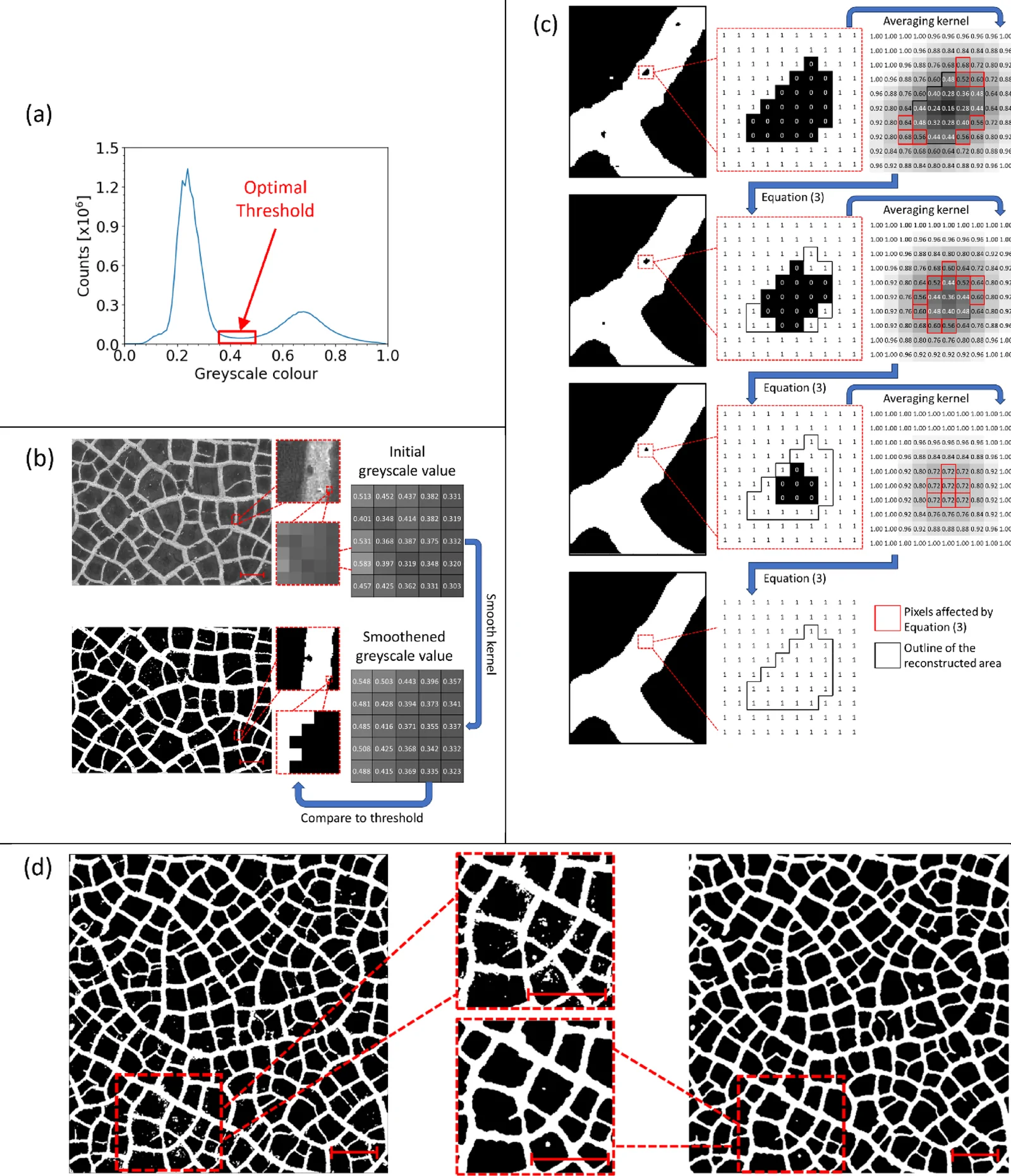
(**a**) Greyscale value count for a random metallic mesh, (**b**) Initial conversion of the picture to binary. The greyscale image is smoothened using the kernel in Eq. (2), and compared to the threshold value (0.42) to produce the pure black and white image. The scalebar is 1 mm, (**c**) Reconstruction steps to eliminate noisy pixels in the body of the network, (**d**) Greyscale to binary conversion of the network after reconstruction. Scale bar is 1 mm.

The colour coefficients are weighted according to human-eye luminance perception^16^, yielding grayscale intensities from 0 (background) to 1 (network). At this stage, branch boundaries remain diffuse due to gradient variations along edges. To discretize the outlines, Otsu’s thresholding^17^ is applied for image binarization. The threshold value is derived from the grayscale histogram^18^, as shown in Fig. 2a, divided into 500 bins to capture intensity distribution. The first histogram peak corresponds to background pixels, the second to network pixels, with intermediate tails representing transition regions. The optimal threshold is selected from the plateau between these peaks, ensuring robust branch-edge definition.

Following threshold determination, a smoothing filter is applied by convolving the image with an averaging kernel, as shown in Eq. (2), of size * N=3*. The averaging kernel reduces the number of pixels with intense colour variation, denoted as “noise” pixels, and aids the edge detection. Pixels with smoothed intensity values exceeding the binary threshold are set to 1; all others are set to 0.

2 $$\:kerne{l}_{smoothing}=\frac{1}{{N}^{2}}\times\:{I}_{N\times\:N}$$

where $\:{I}_{NxN}$ is the identity matrix of size *N*. The resulting binary image, as seen in Fig. 2b, preserves the primary network geometry while retaining minor artefacts from image acquisition.

**Fig. 3:**
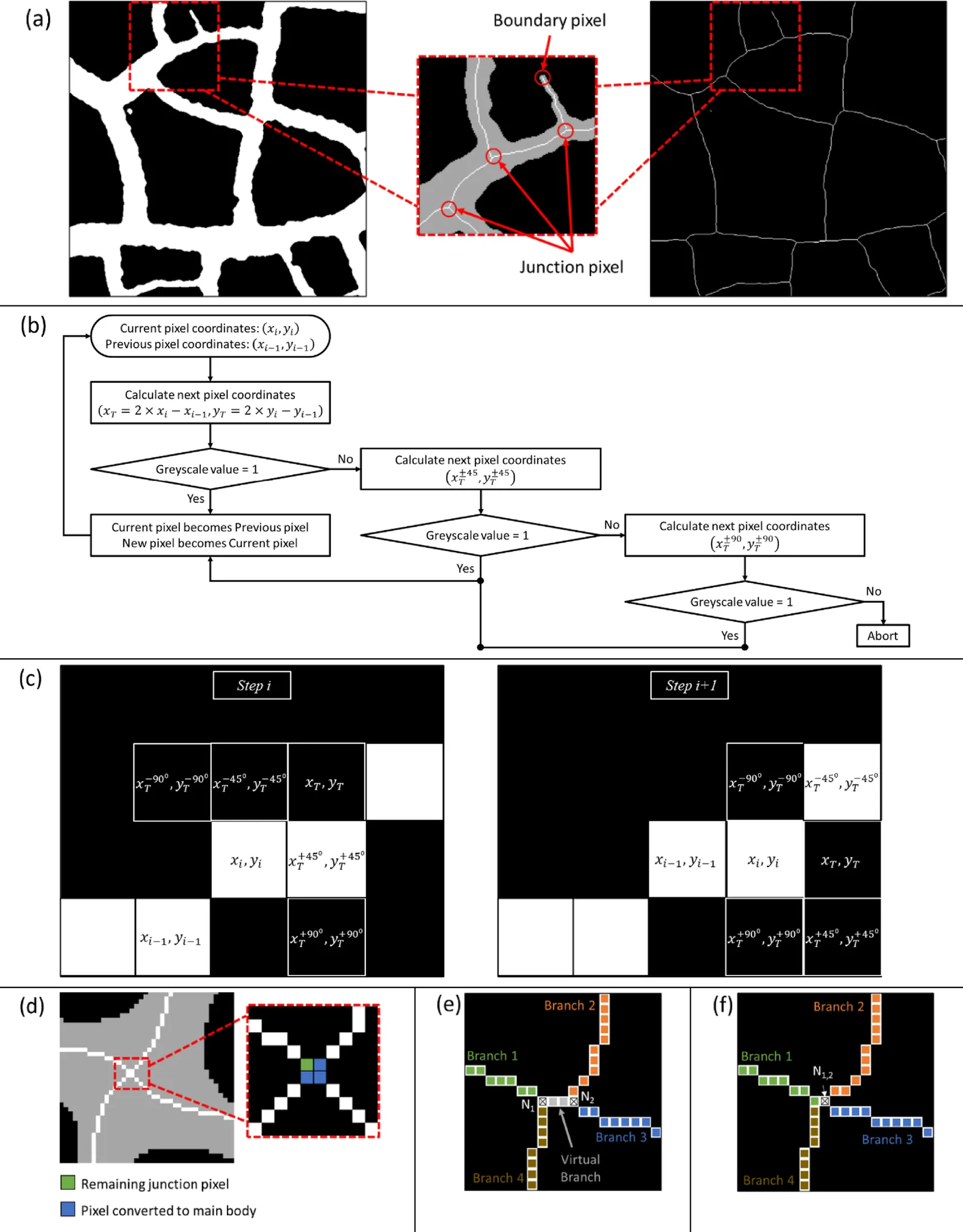
(**a**) Extraction of network’s skeleton using Zhang-Suen’s algorithm, (**b**) Tunnelling method to identify the branches and connect the junction or dangling nodes, (**c**) Example of the branch tracking method at two consecutive iterative steps, (**d**) Resulting cluster of junction pixels from the skeletonisation process, (**e**) Example of a virtual branch between junction nodes N_1_ and N_2_, and (**f**) the resultant local restructure of the skeleton by introducing the junction node N_1,2_ to eliminate the virtual branch.

Residual defects are corrected through an iterative reconstruction filter that compares each pixel to its neighbours. If more than half of the adjacent pixels differ in value, the pixel state is inverted. For computational efficiency, the operation is reformulated using Boolean logic, as seen in Eq. (3). After each iteration, $\:{c}_{\mathrm{recon}}$ replaces $\:{c}_{\mathrm{init}}$; convergence is reached when the change in fill factor, i.e., the ratio of metal coverage, is below 0.01%. As show in Fig. 2c,d, this process effectively reduces noise, smooths pathway contours, and eliminates voids within the network. Any remaining isolated islands (see Fig. 3a) are assessed during the connectivity check, which is described in a subsequent Sect. 

3 $$\:{c}_{recon}={c}_{init}+\left[\left({c}_{smooth}>0.5\right)\wedge\:\left({c}_{init}=0\right)\right]-\left[\left({c}_{smooth}<0.5\right)\wedge\:\left({c}_{init}=1\right)\right]$$

The reconstructed geometry is processed to extract the network skeleton using the skeletonize function from the *skimage.morphology* library, which implements the Zhang - Suen’s algorithm^19^. Pathway width is simultaneously determined via the medial axis transform^20^, computed from the Euclidean distance of each branch pixel to the nearest boundary pixel of opposite colour.

Skeleton pixels are classified as junction, boundary, or main-body pixels. Junction pixels occur at the intersection of three or more branches; boundary pixels terminate incomplete or cut-off branches; all remaining pixels belong to the main body. An example of this classification is shown in Fig. 3a. All skeleton pixels have a binary value of one, with surrounding non-skeletal regions set to zero. Pixel type is determined by summing the binary values of the eight neighbouring pixels: sums of one, two, and greater than two correspond to boundary, main-body, and junction pixels, respectively. Pixels with zero neighbouring values are reclassified as non-skeletal.

The output of the skeletonisation process is a list of key pixels, comprised of the junction and boundary pixels, which would be the initiation/termination points of the branching process. The branching process intends to identify the connecting path between the key pixels and segmentize the network to connected linear elements. A tracking algorithm is used, called tunnelling method, which initiates from a key pixel, and grows the branch pixel-by-pixel, until a new key pixel is met. A schematic of the search sequence can be seen in Fig. 3b and an example of sequence at two iterative steps can be seen in Fig. 3c. Instead of searching all the neighbouring pixels, this method prioritises the pixel that aligns with the local orientation of the branch If the target pixel is not a skeleton pixel, the algorithm looks at the ± 45⁰ orientation, and if unsuccessful, at the ± 90⁰ orientation. If none of the new pixels are skeleton pixels, the algorithm terminates and discards the branch.

**Fig. 4:**
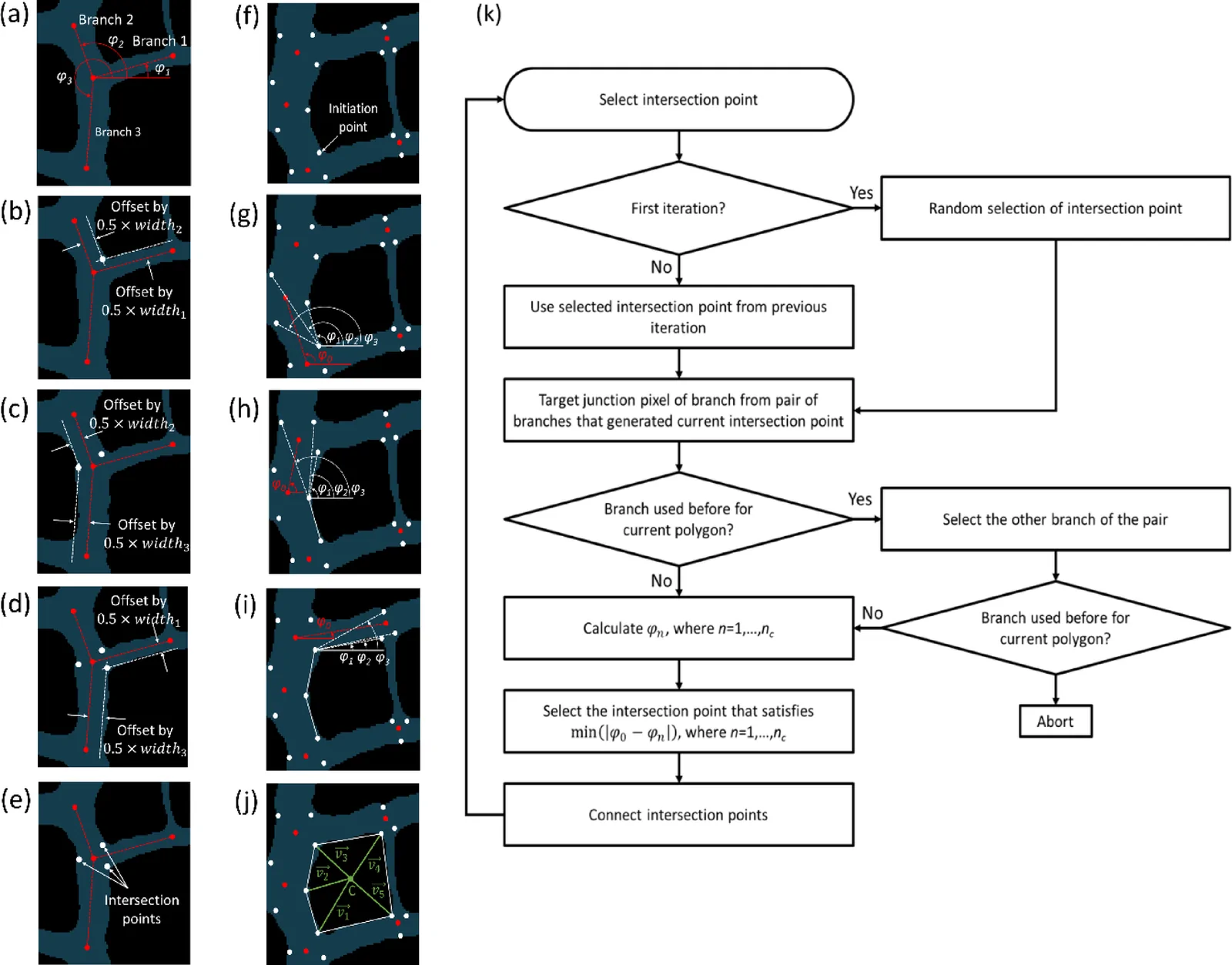
(**a–e**) Calculation of intersection points (white circles) for each valid pair of branches. The red circles represent the junction nodes. The intersection and junction pixels have been magnified (x3) for clarity. (**f–j**) Tracking sequence to identify the valid intersection points and generate the polygon area. (**k**) Tracking sequence algorithm.

As the branch is at its skeletal form, further search around the current pixel is not considered, as the branch cannot change its orientation more than ± 90⁰, as it would indicate a self-folded branch. This approach reduces the number of assessed pixels per iteration from eight pixels to five pixels. Special attention is required for the case where a junction is converted to more than one junction pixels, as show in Fig. 3d.

The generated branches represent conductive pathways with high length-to-width ratios characteristic of crackle geometries. However, skeletonization occasionally produces short, wide branches near junctions that deviate from this ratio. For the meshes studied, a minimum length-to-width ratio of 0.5 ensures over 99% of branches meet this criterion. The remaining “virtual branches” can distort the network’s structure, statistics, and electrical properties. As shown in Fig. 3e a virtual branch only two pixels long and twenty-six pixels wide connects junctions *N*_*1*_ and *N*_*2*_. To correct this, the nodes are merged into a single junction, *N*_*1,2*_, and the associated branches (Branch 1–4) are locally restructured-trimmed and iteratively regrown to connect to the new junction, as seen in Fig. 3e,f.

The successful identification of the branches is followed by the calculation of their width $\:\stackrel{-}{w}$ and length $\:\widehat{l}$ using Eqs. (4) and (5), respectively, using the resolution of the image to translate pixel size to a unit of length. For simplification, a branch is assumed to have uniform width, calculated as the average of the widths $\:{w}_{i}$ of its *n* constituent pixels, although, local width variations, waviness, and constrictions may exist. This approximation reduces geometric complexity and computational cost while preserving the dominant network-scale electro-thermal response. Since Joule heating scales with the square of local current density, averaging branch width may slightly underestimate highly localized peak temperatures in regions containing severe constrictions. However, for the present metallic meshes, temperature distributions are largely governed by global network connectivity, wire density, and heat conduction to the surrounding substrate, such that the effect on the overall thermal response is expected to be small. The ratio of actual length (sum of number of pixels along branch) to estimated straight-line length provides a measure of curvature.

4 $$\:\stackrel{-}{w}=\frac{{\sum\:}_{i=0}^{n}{w}_{i}}{n}\times\:resolution$$

5 $$\:\widehat{l}=\sqrt{{\left({x}_{end}-{x}_{start}\right)}^{2}+{\left({y}_{end}-{y}_{start}\right)}^{2}}\times\:resolution$$

To investigate the effect of this approximation on the network response, a simple calculation, presented in the Supplementary Material, was conducted in which a single branch was treated in two ways. In the first, the branch was modelled as a series of resistors whose wire width varies along its length; in the second, the same branch was assigned a single, uniform width equal to the average of all its segments. Performed for 42 well-resolved branches obtained from high-resolution micrographs, this calculation showed that the simplification underestimates the branch resistance by ≈ 0.3–0.5% (up to ≈ 2% for the rare, strongly necked branch). The uniform-width assumption is thus a deliberate simplification that primarily affects local peak estimates rather than the global or mean response (see Supplementary Figure S2).Other metrics of interest, apart from the width and length of the branch, are the polygon area and the wire density. The polygon area distribution describes network compactness, uniformity, current-path redundancy, and optical transmittance, while wire density quantifies the local concentration of conductive pathways. Higher wire densities generally promote more uniform current distribution and temperature fields, whereas lower wire densities can lead to current localization and an increased likelihood of thermal hotspots^21,22^. Each polygon is defined as a closed loop connecting intersection points, i.e., shared outline pixels of neighbouring branches converging at a common junction, with no intervening branches. Figure 4a,j illustrate the intersection point identification and polygon reconstruction, while Fig. 4k outlines the corresponding workflow. Due to the arbitrary, frequently nonconvex geometries and variable vertex counts, area determination employs vector-based computation. As shown in Fig. 4j, vectors $\:\overrightarrow{{v}_{i}}$ originating from the polygon centroid *C* to each intersection point partition the region into triangular elements. The polygonal area is then obtained as the sum of the cross products of the respective vector pairs.

### Electrical resistance calculation

**Fig. 5:**
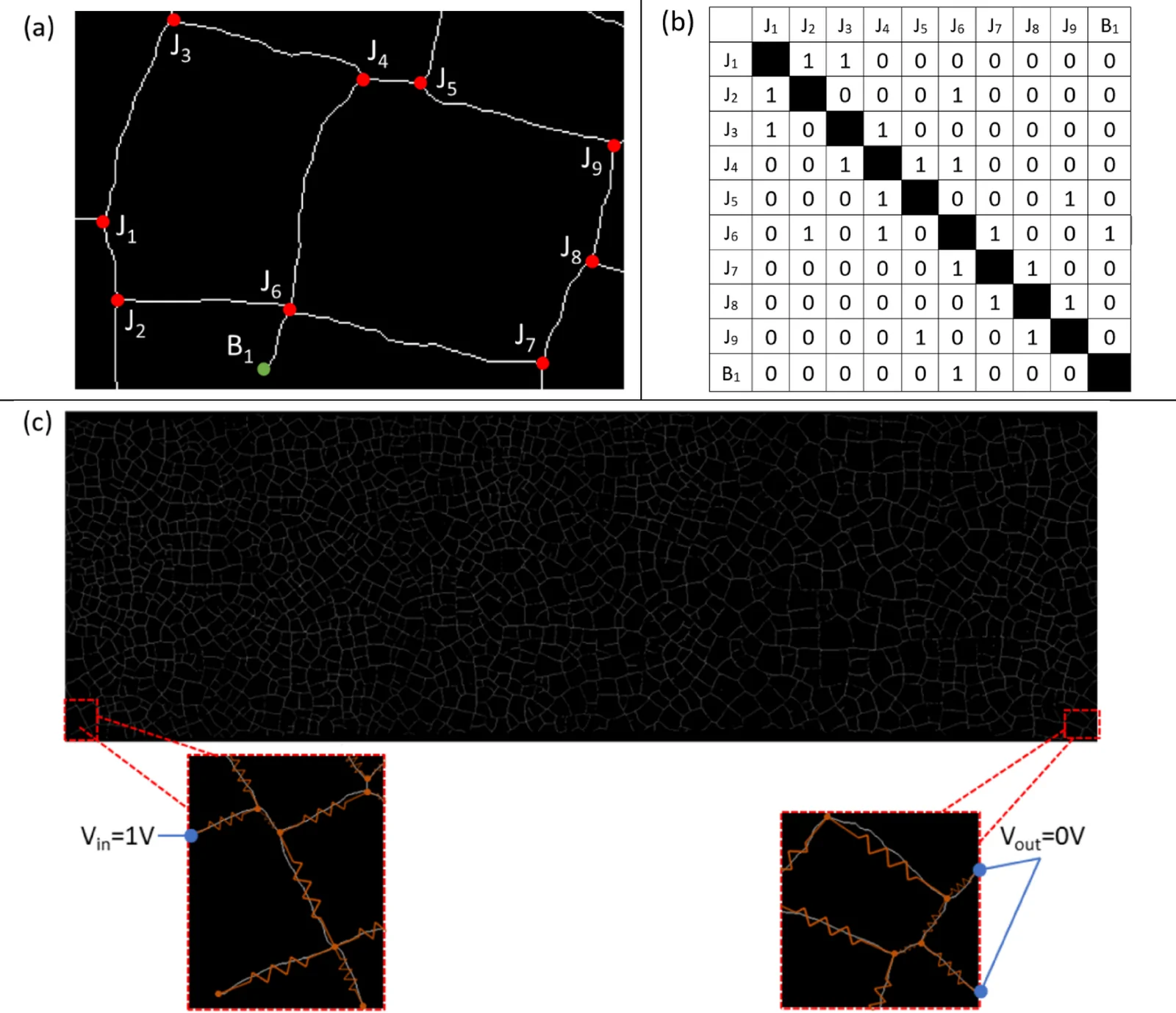
(**a**) Identification of the junction and boundary pixels in a local area of the network, (**b**) Generation of the adjacency matrix to represent the connection of the junction or boundary pixels. ”J” represents a Junction pixel and “B” represents a Boundary pixel. (**c**) Equivalent resistor system of the network’s skeleton and the corresponding electrical boundary conditions.

The branching method formalizes network pathways as line elements enriched with functional attributes. Connectivity among junction and boundary pixels (nodes), is represented via an adjacency matrix derived from graph theory. This matrix encodes nodal affiliations and quantifies connection strength through weights such as length or width. For a network comprising *k* nodes, the adjacency matrix is initialized as a *k × k* zero matrix. Figure 5a,b. present an example for *k = 10*, where each row and column correspond to a node, diagonal entries remain zero (indicating no self-links), and unit values denote existing connections.

The adjacency matrix enables evaluation of branch identification accuracy and quantification of node connectivity, i.e., the number of connections per junction or boundary pixel. Connectivity is obtained as the row sum of the matrix, expected to equal one for boundary pixels and ≥ 3 for junctions; a value of two indicates a misconnected or misclassified region. For the extreme cases of zero connectivity: (i) isolated islands, as shown in Figs. 2d and 3a prior to skeletonization, and (ii) closed loops consisting of only two junction pixels connected exclusively to each other, the algorithm removes the corresponding junction pixels together with the associated isolated feature. The current step is used as a final geometry check to ensure functional connections.

Additionally, the adjacency matrix facilitates computation of the network’s electrical resistance. Binary connections are replaced by weighted values corresponding to the equivalent linear resistors of branches, whose resistances $\:{R}_{i}$ are determined from $\:{R}_{i}={\rho\:}_{e}\times\:{l}_{i}/\left({\stackrel{-}{w}}_{i}\times\:t\right)$, where $\:{l}_{i}$ is the real branch length, $\:{\stackrel{-}{w}}_{i}$ is the average branch width, * t* is the manufacturing-defined thickness, and $\:{\rho\:}_{e}$ is the bulk resistivity. The resulting *k × k* resistor network is formulated as a system of algebraic equations using Kirchhoff’s current law: connection entries are substituted with $\:1/{R}_{i}$, and diagonal terms are updated as the negative sum of each row. The resulting conductance matrix $\:[C{]}_{k\times\:k}$ contains at most $\:{n}_{c}+1$ nonzero elements per row, where $\:{n}_{c}$ denotes the node connectivity. The final formulation is expressed in matrix form in Eq. (6).

6 $$\:{\left[C\right]}_{k\times\:k}\times\:{\left[V\right]}_{k\times\:1}={\left[I\right]}_{k\times\:1}$$

The voltage matrix $\:\left[V\right]$ is obtained by solving Eq. (6), via inversion of the conductance matrix $\:\left[C\right]$. Matrix $\:\left[C\right]$ must be full-rank and diagonally nonzero to ensure network connectivity. Disconnected branches are removed by deleting their rows and columns, and parallel branches between identical nodes are reduced to equivalent resistances. Boundary conditions are applied to the current vector $\:\left[I\right]$, as can be seen in Fig. 5c, as $\:{V}_{\mathrm{in}}/R$, with $\:{V}_{\mathrm{in}}=1 V$; opposing terminals are grounded.

The solution yields nodal potentials and corresponding input currents, from which total current and network resistance are derived. This resistor-network formulation enables evaluation of disconnections and manufacturing tolerances by adjusting branch dimensions or nullifying elements, allowing estimation of resistance variability rather than a single deterministic value.

### Experimental methods

Two sample types were prepared. The first consisted of a single Polyethylene Terephthalate (PET) layer interfaced with a random silver network (“Interlayer”). The second comprised the Interlayer embedded within a Polycarbonate (PC) – Poly-vinyl Butyral (PVB) – Interlayer – PVB – PC stack (“Laminated”). PVB was supplied by Pilkington; PC and PET were obtained from Goodfellow.

Metallic meshes with different wire densities were fabricated via physical vapour deposition of silver onto crack patterns formed during drying of colloidal dispersions. The fabrication of the meshes and sample assemblies is described in Qiang et al.^23^. For all samples, 50-µm thick brass strips were embedded along each side to ensure uniform electrical contact; the brass thickness was selected to match the mesh, as thicker brass caused tearing due to thermal-expansion and size mismatch. Reference dimensions for each sample type are listed in Table 1.

Electrical resistance was measured using a Keithley 2100 (6½-digit) multi-meter. Electrothermal performance of Interlayer samples was assessed with a FLIR A6750 IR camera at 120 fps under static voltage. Mechanical performance of laminated samples was evaluated via three-point bending tests (ASTM D790-03, Procedure A) using an Instron 68FM100, with a 51.2 ± 0.1 mm support span, 4.4 ± 0.1 mm overhang, and a crosshead rate of 120 ± 0.1% mm/min. Rollers were insulated to prevent electrical conduction. Sample temperature was checked with a non-contact thermometer before testing.

All tests were performed at 22 °C. A maximum current of 3 A was supplied through the brass contacts. The chosen loading sequence was: (i) application of electrical current for two seconds (pre-heating stage), (ii) initiation of steady-state deflection for one second, resulting in a total of 2 mm deflection, while maintaining the electrical current (loading stage). Each experiment was repeated three times, and an average was calculated.

**Table 1:** Sample dimension for the current study.

Sample type	Thickness ± 10%				Length ± 0.5 (mm)	Width ± 0.1 (mm)	Sample thickness ± 0.1 (mm)
	PVB (mm)	PET (mm)	PC (mm)	Mesh (um)			
Interlayer	–	0.17	-	0.75	60.0	15.0	0.17
Laminated	0.38	0.17	1.00	0.75	60.0	15.0	2.90

### Numerical modelling

**Fig. 6:**
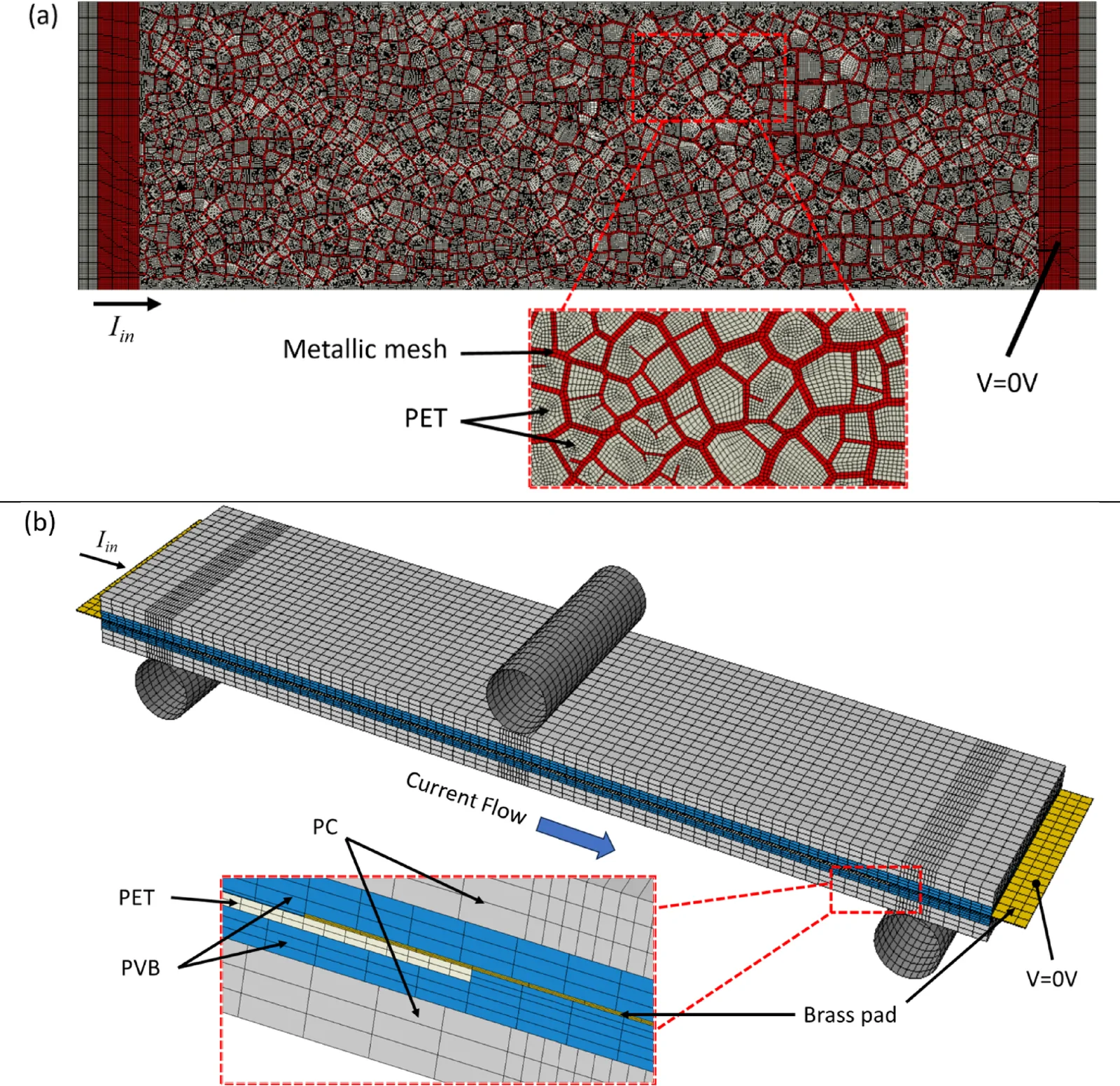
(**a**) Numerical models used for the electro-thermal simulation of the Interlayer model. (**b**) Numerical models used for the electro-thermo-mechanical simulation of the Laminated model.

The finite element method (FEM) was used to simulate the electro-thermo-mechanical behaviour of the samples in Abaqus/Explicit 2020. Interlayer samples were analysed with a coupled thermal-electrical model (two degrees of freedom (DOF): voltage, temperature), while Laminated samples use a fully coupled electro-thermo-mechanical model (five DOF: 3 displacements, voltage, temperature). The electro-thermal model, see Fig. 6a, uses linear coupled thermal-electrical brick elements (DC3D8E). The fully coupled model, see Fig. 6b, uses 8-node brick elements with reduced integration and hourglass control (Q3D8R). Substrate regions interfacing with the metallic mesh are partitioned to improve mesh transitions, enable hex-dominated meshing, and optimise network–substrate interaction. Additional partitioning and refinement are applied near mechanical contact regions (see Fig. 6b). All materials (metallic mesh, PC, PVB, PET, brass pads) are modelled as elastic, with properties listed in the supplementary material (see Table S – 1).

The computational efficiency of the framework arises primarily from topology-preserving simplification of the image-derived geometry, whereby high-resolution pixel morphology is replaced by equivalent branch representations characterised by length, width, and connectivity. This reduces preprocessing complexity and improves mesh generation robustness while preserving the physical pathways governing electro-thermal transport.

The Interlayer model is created from the digitised random mesh geometry, extruded to 3D, and meshed mainly with hexahedral elements of size ~ 0.08 mm (≈ 1 st quartile of wire width), yielding ~ 250,000 elements (Fig. 6a). The Laminated model uses a similar approach and contains ~ 360,000 elements (Fig. 6b). Rollers were modelled as rigid shells, with the bottom rollers fully fixed and the top roller constrained in the perpendicular direction. Frictional contact (*µ* = 0.12) is defined between rollers and structure. Current and ground voltage are applied to the brass pad surfaces.

PET is modelled as linear elastic without viscoelasticity, as its glass transition temperature (~ 104 °C) exceeds the experimental temperature range (< 70 °C). In contrast, PVB exhibits a glass transition near 31.6 °C, meaning PET remained in its glassy state while PVB underwent glass transition (see Supplementary Material Figure S – 1).

A convection coefficient of 10 W/m²K was applied to the polymer substrate surfaces, consistent with natural convection in still air. A larger coefficient of 35 W/m²K was assigned to the exposed metallic network; similar approaches have been adopted in electro-thermal modelling of wire-based and network-based heating structures, where enhanced heat dissipation from the metallic pathways is observed relative to the surrounding substrate^24^.

Compared with direct image-derived finite element discretisation approaches explored in our previous work^25^, the present topology-preserving simplification substantially reduces model complexity. Specifically, the effective number of DoF was reduced from approximately 20 million to approximately 1.8 million while retaining the extracted network topology and electro-thermal predictive capability. This reduction arises from replacing pixel-level geometric detail with equivalent branch representations characterised by width, length, and connectivity, thereby improving mesh generation robustness, and enabling tractable coupled multiphysics simulations.

## Results and discussion

### Network characterisation

**Fig. 7:**
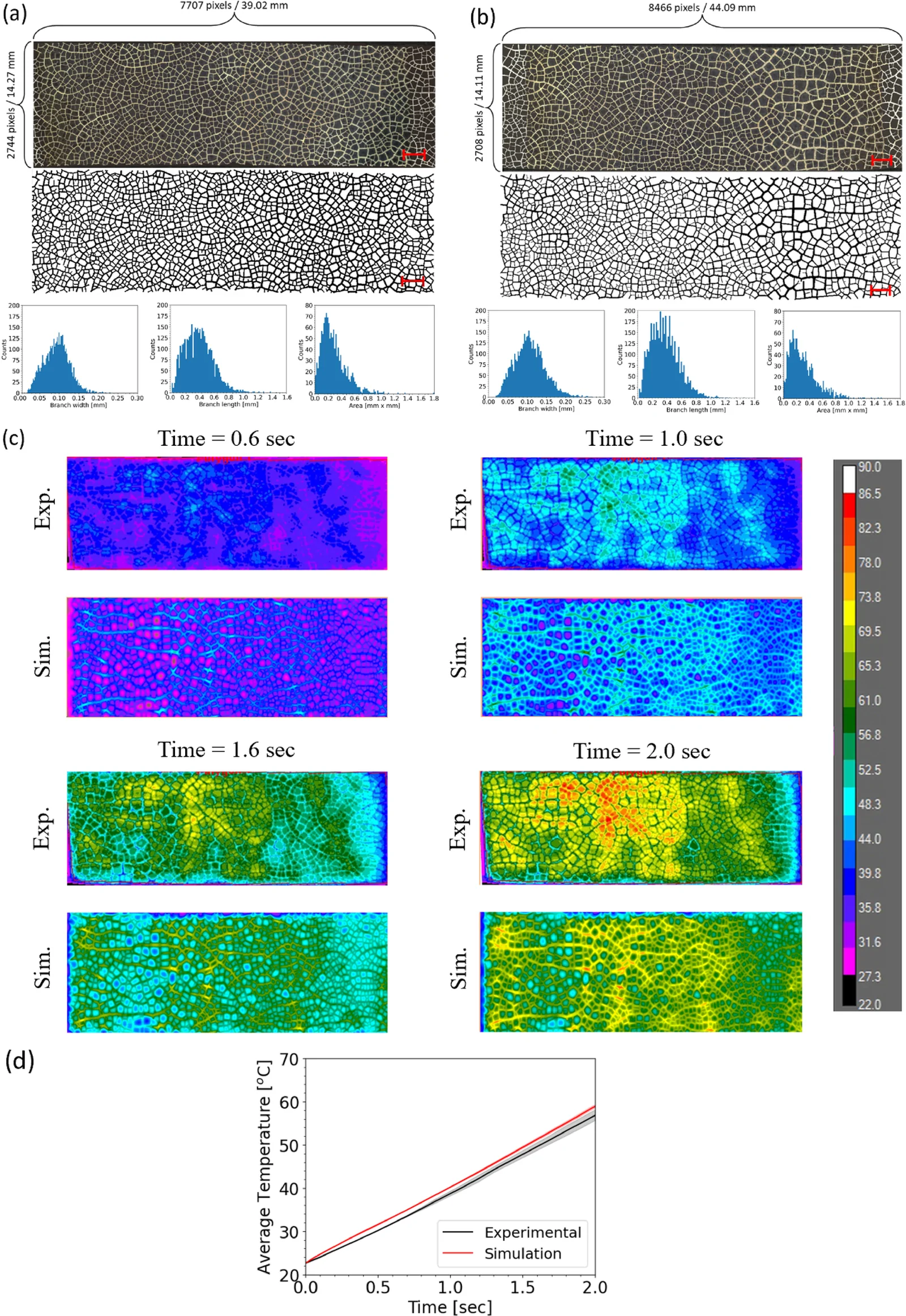

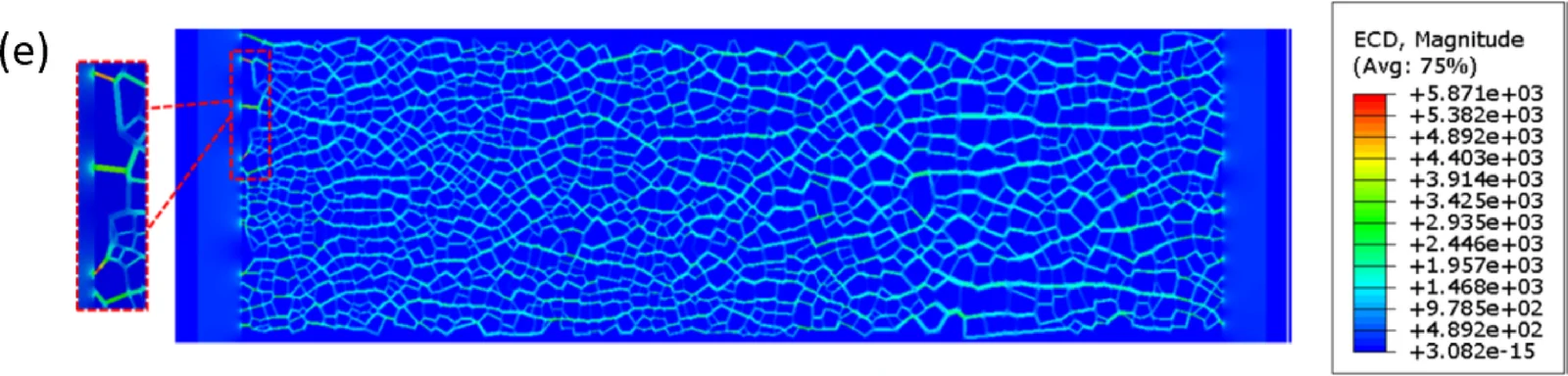
(**a**) Digitisation of SM1. The image captured by the microscope(above), the generated simplified geometry (middle), and the distributions of the branch width and length and polygon area (bottom). This image has approximately 20.5 million pixels. The scalebar is 2 mm. (**b**) Digitisation of SM2. The image captured by the microscope (above), the generated simplified geometry (middle), and the distributions of the branch width and length and polygon area (bottom). This image has approximately 23 million pixels. The scalebar is 2 mm. (**c**) Comparison of the developed temperature field between experimental and numerical analysis results. The units of the colour bar are **°**C (**d**) Mean value of average temperature recorded for networks under 3 A. The shaded area represents the spread of recorded values from different samples. (**e**) Electrical Current Distribution (ECD) at 3 A excitation. The units of the colour bar are A/mm^2^.

Two metallic meshes, *SM1* and *SM2*, were analysed. Their branch-width and branch-length distributions, as well as polygon-area distributions, are shown in Fig. 7a,b, respectively. Characterisation data and summary statistics are presented in Tables 2 and 3, respectively. Because the distributions are skewed, summary statistics are reported using the first three quantiles rather than mean and standard deviation. Skewness (Pearson–Fisher) and kurtosis (Fisher-adjusted) are also included to describe distribution asymmetry and tail behaviour. The digitisation process exhibits right-skewed distributions consistent with literature^26^.

Electrical resistance of at least four samples was measured using a two-probe setup, with probes contacting side pads in ohmic contact with the network. Measured resistance * R* was converted to sheet resistance using $\:{R}_{sheet}=R\times\:\left(W/L\right)$, where *L* is the length of the network (along current flow), and *W* is the width of the network (lateral to current flow). Sheet resistance is reported in Ω/sq. (ohms per square), a conventional notation indicating the resistance of a square section of thin film independent of its lateral dimensions. The average equivalent sheet resistance of *SM1* and *SM2* was 0.19 ± 0.01 Ω/sq. and 0.18 ± 0.01 Ω/sq., respectively. The analytical model presented in the current study yielded 0.185 Ω/sq. and 0.177 Ω/sq., in close agreement with experiment, confirming accurate digitisation of network geometry and connectivity.

**Table 2:** Outer dimensions, fill factor, wire density, number of junctions and average connectivity of *SM1* and *SM2*.

Mesh	Length [mm]	Width [mm]	Thickness [nm]	Fill factor	Wire density [wires/mm^2^]	Total number of junctions	Average connectivity
*SM1*	39.02	14.27	750	26.28%	7.67	2673	3.06
*SM2*	44.09	14.11	750	28.56%	6.92	2658	3.08*

*SM2 was the only digitised network that showed a connectivity of 5 on a junction.

**Table 3:** Summary statistics of the branch width and length, and polygon area of *SM1* and *SM2*.

Mesh	Branch width [µm]					Branch Length [µm]					Polygon Area [mm^2^]				
	1QT	2QT	3QT	Skew	Kurt	1QT	2QT	3QT	Skew	Kurt	1QT	2QT	3QT	Skew	Kurt
*SM1*	71	95	115	0.25	0.15	229	349	479	0.72	1.18	0.16	0.25	0.39	1.58	4.83
*SM2*	80	105	131	0.51	0.57	239	365	505	0.88	1.54	0.15	0.26	0.41	2.05	10.05

### Electro-thermal performance

To further assess the ability of the proposed framework to capture the spatial electro-thermal response of the network, experimentally measured infrared thermal images obtained from the Interlayer sample were compared with the corresponding numerical temperature fields at selected time intervals during Joule heating, as shown in Fig. 7c. The performance was evaluated under a constant 3 A current, applied for a maximum of 2 s to prevent local overheating. Recorded temperatures represent the combined area of the metallic network and exposed substrate.

The experimental measurements and numerical predictions were in reasonable agreement, exhibiting a similar evolution of the temperature field, and the model reproduced the formation and propagation of the warmer regions observed experimentally. The principal discrepancy was a more diffuse measured field, characterised by a broader distribution of elevated temperatures than that obtained numerically. This broadening is not attributable to the uniform-width representation, which affects only the simulation and, by neglecting local constrictions, would tend to render the simulated field smoother rather than more diffuse. It is instead associated with measurement- and modelling-related factors: principally the finite spatial resolution and accuracy of the infrared thermography, which averages over sub-resolution features, and the simplified representation of convective heat loss. In the latter, convection coefficients were adopted from the literature, and local variations in airflow and heat dissipation not captured by these values may produce a wider spatial spreading of heat than predicted. Despite these limitations, the simulation accurately identified the principal hotspot locations and predicted temperature levels that were in good agreement with the experimental observations.

Figure 7d compares the average temperature evolution of the metallic network obtained experimentally with the corresponding numerical predictions. After 2 s of electrical activation, the mean temperature increased by approximately 34.2 °C in the experiments and 36.3 °C in the simulations, corresponding to heating rates of 17.1 °C/s and 18.2 °C/s, respectively. Figure 7e presents the predicted current-density distribution, which remains relatively uniform throughout most of the conductive network, indicating efficient current transport and the absence of significant current bottlenecks. Together, these results demonstrate that the proposed framework can accurately predict both the spatial and temporal electro-thermal response of the conductive network, providing confidence in the validity of the electro-thermal model.

The measured heating rates here are substantially higher than those reported in our previous studies on embedded metallic heating structures using one-dimensional and two-dimensional regular mesh architectures^27,28^. This improvement can be attributed to the morphology of the heating network. The thermal response is strongly influenced by the characteristic dimensions of the heating elements and the network fill factor^26^. Template-assisted manufacturing enables the formation of junction-free interconnected metallic networks with branch diameters of a few micrometres. The absence of junction resistance, combined with the highly interconnected architecture, enhances electrical conductivity and promotes more efficient Joule heating compared with conventional regular mesh designs. This is relevant for thermally activated stiffness modulation, as faster heating enables more rapid temperature increase within the interlayer and therefore accelerates the softening response of the PVB phase. Consequently, the present metallic mesh architecture supports faster electro-thermal activation and reduced response times for de-stiffening applications.

Beyond predicting global heating behaviour, the framework also provides insight into localized electro-thermal phenomena. The ability to identify both the overall current distribution and local temperature concentrations is important for transparent heating structures, where localized overheating might not necessarily affect the global macroscopic metrics such as mean temperature or global force-deflection curves, but it can certainly lead to material degradation or failure^27^. Consequently, the framework provides a practical tool for assessing thermal safety margins while enabling investigation of topology-dependent phenomena such as local current redistribution, hotspot formation, and the influence of network features. This advantage is most evident in networks with a low fill factor, large branch-width variations, or weak conductive coupling, where local current crowding and hotspot formation dominate the electrothermal response. These local effects cannot be captured by a homogenised effective-medium model. In contrast, for the well-formed, high-fill-factor meshes studied here, the current and temperature fields remain largely uniform, so a homogenised model would predict the mean response with similar accuracy. The role of such manufacturing-induced features is illustrated in the Supplementary Material, where a localised defect produces a hotspot (Figure S3) and a gradient in mesh topology produces a corresponding temperature gradient (Figure S4).

### Electro-thermo-mechanical performance

**Fig. 8:**
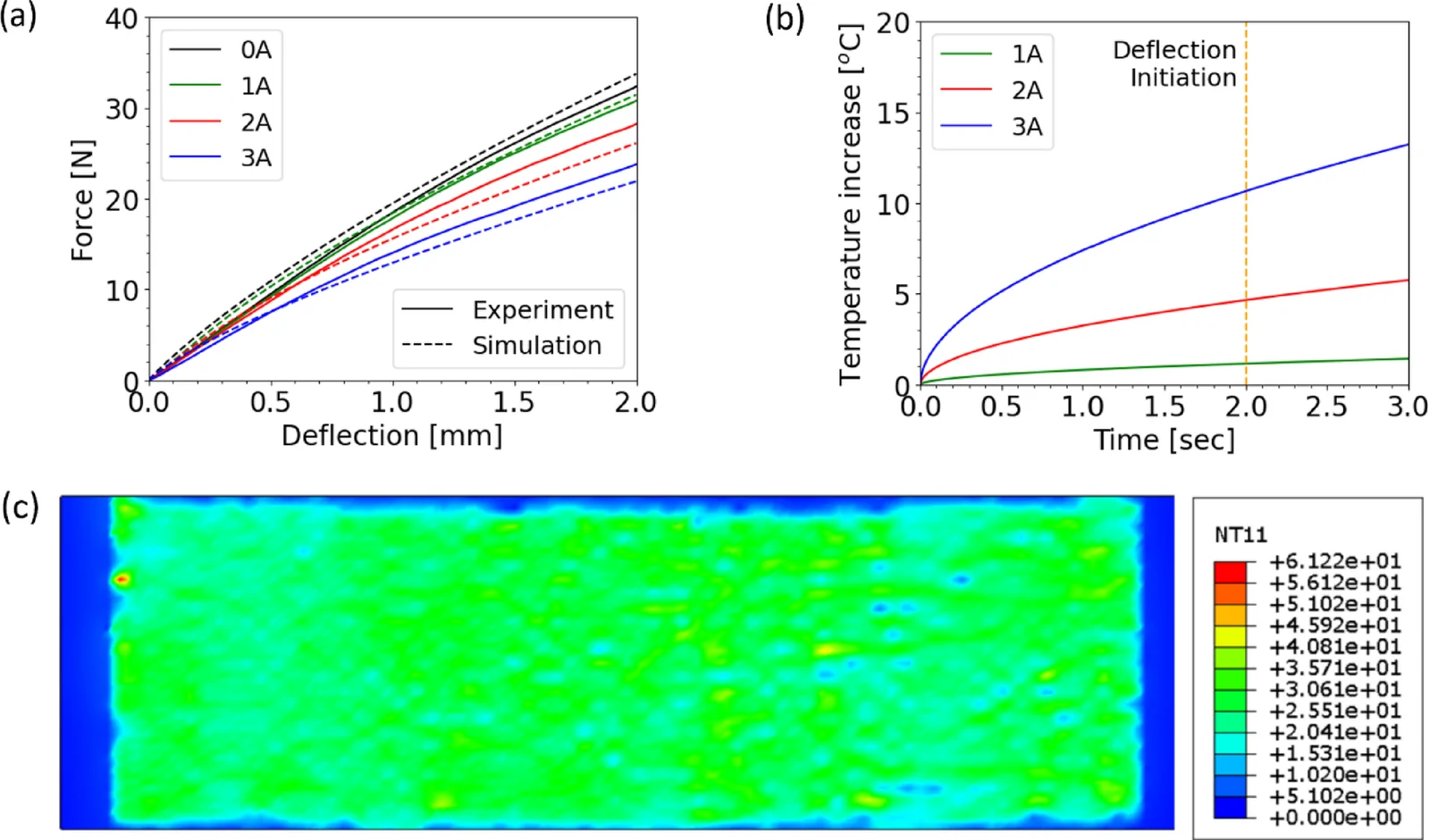
(**a**) Electro-thermo-mechanical performance of Laminated samples and comparison with the numerical model using the digitised mesh. (**b**) Temperature increase attained on the interlayer based on numerical results. (**c**) Nodal Temperature (NT) distribution of the interlayer of the Laminated sample under 3 A of current excitation. The units of the colour bar are °C.

Force–deflection curves for different current levels are shown in Fig. 8a. Experimental and numerical results agree closely. Numerical models were further used to estimate interlayer temperatures, shown in Fig. 8b, with corresponding reductions in reaction force and temperature increases summarised in Table 4. The induced temperature field at 3 A (Fig. 8c) is largely uniform with minimal hotspots. The PET layer remained below 45 °C, which is well under its glass-transition temperature, confirming that PET stays in the glassy regime and justifying the linear-elastic assumption.

Although current was applied longer here than in the electro-thermal tests (3 s vs. 2 s), hotspot temperatures were lower. This is attributed to dominant conductive heat loss (small Biot number). The Laminated configuration increases the network–polymer contact area, enhancing conduction and reducing heat retention in the mid-plane. These observations, together with the numerical temperature profiles, validate the choice of a 3-second excitation, ensuring full PVB transition with minimal damage to the heating element.

**Table 4:** Experimental and numerical results of the electro-thermo-mechanical behaviour of the Laminated samples and the numerically evaluated attained temperature increase on the sample’s interlayer.

Electrical current (A)	Experimental flexural modulus (MPa)		Force at 2 mm deflection (*N*)		Temperature increase after 3 s (⁰C)
	0–0.2 mm	1.8–2 mm	Experiment	Numerical	Numerical
0	1334.2	901.4	32.4	33.7	–
1	1298.1	829.3	30.8	31.5	1.4
2	1161.9	695.1	28.1	26.1	5.8
3	1052.8	667.1	23.8	21.9	13.2

### Effect of dangling branches

To further reduce geometric complexity and computational cost, dangling branches, segments not contributing to electrical conduction, were removed. Although electrically inactive, these branches slightly enhance heat transfer by increasing the mesh–polymer contact area.

**Fig. 9:**
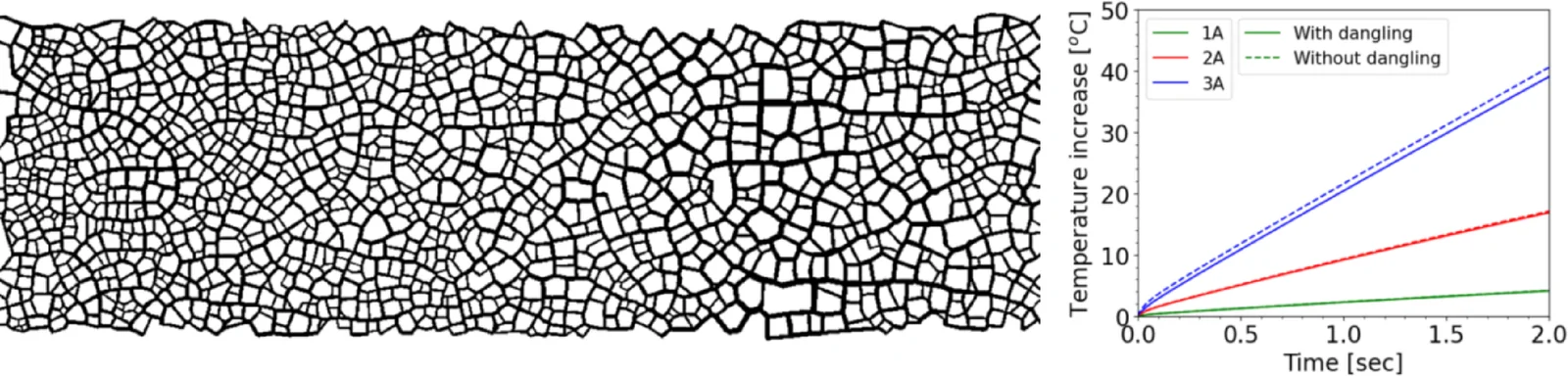
Simplified SM2 metallic network without dangling branches and the effect of the exclusion of dangling branches on the electro-thermal behaviour at different current excitations.

The simplified *SM2* geometry is shown in Fig. 9. Branches on the left and right edges were retained because they connect directly to the source and ground. The Interlayer numerical model was used to evaluate electrical and electro-thermal performance of the simplified network under 1, 2, and 3 A excitation. Removing dangling branches reduced total network elements by 4% and wedge elements by 6%, improving mesh quality. As expected, electrical resistance remained unchanged. Average temperature increase (Fig. 9) was nearly identical between the full and simplified model for 1 A and 2 A, and differed by less than 2 °C at 3 A. Because the temperature fields were highly similar, full electro-thermo-mechanical simulations were not conducted. Temperature and current distributions closely matched those of the full model (see Fig. 7d,e for comparison). Temperatures were slightly higher (≈ 2 °C) due to reduced conductive heat-exchange area. In this small-scale network, the influence of dangling branches is minimal; however, their impact is expected to increase in networks with comparable pathway dimensions but lower fill factors.

## Conclusions

The current work presented a computationally efficient framework to systematically investigate the electrical, electro-thermal, and electro-thermo-mechanical behaviour of structures incorporating random metallic meshes as heating elements. The developed digitisation and geometry-simplification algorithm enabled efficient numerical modelling, which showed strong agreement with experimental results. An analytical method for predicting sheet resistance directly from network images was also integrated into the workflow. Key findings are:

- The developed algorithm provides an automated tool to accurately capture network geometry, providing in strong agreement between the predicted electrical, electro-thermal and electro-thermo-mechanical behaviour and the experimental values.
- The presented computational framework improves the tractability of image-derived multiphysics modelling by reducing model complexity through topology-preserving simplification. Relative to direct image-derived discretisation explored in previous work, the effective number of DoF decreased from approximately 20 million to 1.8 million while maintaining predictive accuracy for electro-thermal and electro-thermo-mechanical behaviour.
- The analytical method implemented in the framework provides statistical metrics and the sheet resistance of the network within a minute with reliable accuracy, offering critical information for the performance of transparent heaters and smart electronics, e.g., potential hotspots, current distribution.
- The developed algorithm enables the study of network features, e.g., dangling branches, and their impact on the network’s performance, which is crucial for applications such as transparent thermal heaters. Such algorithms would greatly facilitate the exploration of the design space when coupled with automated network generation methods.

## Supplementary Information

Below is the link to the electronic supplementary material.

Supplementary Material 1

## Data Availability

The datasets generated and/or analysed during the current study are available from the corresponding author on reasonable request.
