# Supplementary material for: Image-based multiphysics modelling of random metallic meshes
Source: Sci Rep. 2026 Jul 28;16:34173. doi: 10.1038/s41598-026-62432-3 (PMC13710194; doi:10.1038/s41598-026-62432-3)
Supplement: Supplementary file 1 [file 41598_2026_62432_MOESM1_ESM.docx]

# Supplementary material

## Material properties for the numerical modelling

Table S - 1. Properties for the simulated materials. Temperature-dependent materials are expressed as functions of temperature T.

| Material | Density  [kg m^-3^] | Electrical conductivity  [S m^-1^] | Thermal conductivity  [W m^-1^ ⁰C^-1^] | Elastic modulus [GPa] | Specific heat  [J kg^-1^ ⁰C^-1^] | Poisson’s ratio |
| --- | --- | --- | --- | --- | --- | --- |
| PET ^1,2^ | 1300 | ${10}^{-16}$ | 0.2 | 3 | 1200 | 0.43 |
| Silver^3–6^ | 10500 | $\frac{61.4\times{10}^{6}}{1+0.0041\times\left( T-20 \right)}$* | 429 | 82.7 | $235.3+0.0863\times T$ ** | 0.37 |
| Brass^6–8^ | 8450 | 15.9×10^6^ | 120 | 110 | 380 | 0.34 |
| PC ^6^ | 1200 | 10^-12^ | 0.2 | 2.315-0.00522×T | 1200 | 0.37 |
| PVB^9^ | 1080 | 3.2×10^-12^ | 0.2 | see Table S - 2 | $1832+2.62\times T$ ** | 0.47 |

*The electrical conductivity is calculated based on $\sigma=\frac{\sigma_{0}}{1+\alpha\times\left( T-T_{0} \right)}$, where σ_0_ is the resistivity at 20⁰C, α is the temperature coefficient of resistance, and *T_0_* is the reference temperature.

**The specific heat equation is linearly fitted in the experimental data presented in the referenced publications.

The elastic modulus $E_{\left( t \right)}$ for PVB was modelled using the following equation:

| $E_{\left( t \right)}=E_{\infty}+\sum_{i=1}^{n} E_{i}\times e^{-\frac{t}{\tau_{i}}}$ | (S - 1) |
| --- | --- |

where $E_{\infty}$ is the long-term elastic modulus, $E_{i}$ is the elastic modulus associated with the relaxation time, and $\tau_{i}$ is the relaxation time. The materials were assessed using DMA and the results were used to calibrate the viscoelastic parameters $E_{i}$ and the relaxation time $\tau_{i}$. The values can be found in Table S - 2.

Table S - 2. Viscoelastic parameters and relaxation times for the characterisation of the mechanical properties of PVB. The instantaneous modulus $E_{0}=E_{\infty}+\sum E_{i}$ and $E_{\infty}=0.5MPa.$

|  | $E_{i}/E_{0}$ | $\tau_{i}$ |  | $E_{i}/E_{0}$ | $\tau_{i}$ |
| --- | --- | --- | --- | --- | --- |
| 1 | 3.72E-01 | 1.00E-09 | 9 | 6.38E-02 | 1.00E-01 |
| 2 | 1.43E-15 | 1.00E-08 | 10 | 4.07E-02 | 1.00E+00 |
| 3 | 9.15E-02 | 1.00E-07 | 11 | 1.54E-02 | 1.00E+01 |
| 4 | 7.18E-02 | 1.00E-06 | 12 | 5.26E-03 | 1.00E+02 |
| 5 | 7.97E-02 | 1.00E-05 | 13 | 6.04E-04 | 1.00E+03 |
| 6 | 8.52E-02 | 1.00E-04 | 14 | 3.74E-04 | 1.00E+04 |
| 7 | 8.98E-02 | 1.00E-03 | 15 | 1.94E-04 | 1.00E+05 |
| 8 | 8.33E-02 | 1.00E-02 | 16 | 2.29E-04 | 1.00E+06 |

The PET is modelled as linear body without viscoelastic properties due to its glass transition temperature being greater than the attained temperature profiles in the experiments. A comparison of the tan delta between the PVB and PET can be seen in Figure S - 1. The peak of the graphs designates the glass transition area for each material. PVB exhibits a peak close to 31.6⁰C, and PET close to 104.1⁰C. The high glass transition temperature of PET, combined with the studied temperature range in the experiments (< 70⁰C) signifies that PET is in its glassy state, while PVB experiences glass transition.

Figure S - 1 Tan delta of PET and PVB with the corresponding glass transition temperature.

## Within-branch width variation and the uniform-width approximation

The framework assigns each branch a mean width. The variation between branches is therefore retained, and only the variation of width along each branch is discarded by the uniform-width approximation. To quantify the resulting error, the width profile $w_{\left( l \right)}$ along each branch was calculated from the high-resolution microscopy using the medial-axis (distance) transform, which returns the local half-width at every point along the branch skeleton. For each of 42 well-resolved branches the resistance was then evaluated in two ways. In the first way, the branch was treated as a series of elementary resistors of local width *w(l)*, giving the resolved branch resistance

$$R_{Variable}=\frac{\rho}{t}\times\int_{0}^{L} \frac{dl}{w_{\left( l \right)}}$$

*(S1)*

*,*

while in the second way, it was assigned its mean width *w̄*, giving the uniform-width branch resistance estimate

$$R_{Uniform}=\frac{\rho}{t}\times\frac{L}{\overline{w}}$$

*(S2)*

where *ρ* is the bulk resistivity, *t* the film thickness and *L* the branch length. The branch resistance depends on $1/w$, and its width *w* can be expressed in terms of its mean value and the local deviation as $w=\overline{w}+\delta$. Using a second-order Taylor expansion about the mean width yields:

$$\frac{1}{w}=\frac{1}{\overline{w}}-\frac{\left( w-\overline{w} \right)}{\left( \overline{w} \right)^{2}}+\frac{\left( w-\overline{w} \right)^{2}}{\left( \overline{w} \right)^{3}}$$

*(S3)*

The term $w-\overline{w}$ can be substituted by $\delta$, based on its definition above. This substitution simplifies Eq. (S3) into:

$$\frac{1}{w}=\frac{1}{\overline{w}}-\frac{\delta}{\left( \overline{w} \right)^{2}}+\frac{\delta^{2}}{\left( \overline{w} \right)^{3}}$$

*(S4)*

Substituting Eq. (S4) to Eq. (S1), and given that $\left\langle\delta\right\rangle=0$ and $\left\langle\delta^{2} \right\rangle/\left( \overline{w} \right)^{2}=CV^{2}$, where *CV* is the coefficient of variation of *w* within the branch, Equation S1 can simplified to:

$$R_{Variable}=\frac{\rho}{t}\times\frac{L}{\overline{w}}\times\left( 1+CV^{2} \right)$$

*(S5)*

Direct comparison of the electrical resistance of the branch using the two methods yields:

$$\frac{R_{Variable}}{R_{Uniform}}=1+CV^{2}$$

*(S6)*

The uniform-width treatment therefore always slightly underestimates the branch resistance, by an amount set by the square of the within-branch width dispersion.

Across the 42 branches the within-branch width was found to vary only weakly, with a median CV ≈ 0.06 (Figure S2). The corresponding per-branch resistance underestimate is only ≈ 0.3–0.5% on average, rising to ≈ 2% for the small number of branches that contain a pronounced local constriction. The same values are obtained from the direct resolved-versus-uniform calculation [Eqs. (S1) and (S2)] and from the second-order estimate of Eq. (S6), confirming the consistency of the two approaches.

Figure S2. Within-branch width variation measured from the microscopy. Five branches traced on the digitised mesh and their width profiles along the branch length: the width is nearly flat within a branch (small CV), whereas the large differences are between branches.

.

The approximation has a larger, though still modest, effect on local quantities. The current density in a branch is highest where the branch is narrowest, according to:

$$J=\frac{I}{w\times t}$$

*(S7)*

This local maximum is not captured when the branch is represented by its mean width. The associated heat generated via Joule heating, per unit volume is:

$$Q=\rho\times J^{2}$$

*(S8)*

The expression in Eq. (S8) can be further reworked using the same process as described for Eq. (S3) - (S5), resulting in:

$$\frac{Q_{Variable}}{Q_{Uniform}}=1+3\times CV^{2}$$

*(S9)*

Using the *CV* values from Figure S2, it is evident that for the extreme value of *CV*=0.14, the simplification error in the branch-integrated Joule heating is below $\sim$6%. In the connected network a constriction is a high-resistance site, so current redistributes through parallel branches and reduces the local concentration; in addition, the heating element is strongly coupled to the substrate by conduction (small Biot number), which smears any local heating in the resulting temperature field, so the effect on the peak temperature is smaller still.

At the network scale the effect is correspondingly small. Independently of the per-branch analysis, the sheet resistance predicted by the analytical model, which uses the same uniform-width assumption, agrees with the measured value to within ≈ 3%, bounding the net influence of the approximation on the global response. The uniform-width assumption is therefore a deliberate simplification that primarily affects local peak estimates rather than the global or mean response.

## Non-destructive identification of manufacturing defects and local hot-spot formation

The metallic meshes presented in the main manuscript (SM1 and SM2) were selected as examples of high-quality manufacturing, in which the crackle network forms uniformly, and the resulting temperature field is correspondingly uniform. In practice, the deposition process can also produce localised geometric imperfections, in the form of pronounced branch-width variation and constrictions, or regions of low network density and voids, that are not always evident from a global resistance measurement. Because the image-based framework resolves the local geometry directly from microscopy, these features can be identified non-destructively, before they manifest as device failure. Figures S3 and S4 illustrate this on samples that exhibit such manufacturing variability.

Figure S3. A locally damaged / low-density region of the mesh and its electro-thermal consequence. Top: micrograph of the strip, showing a localized void / region of missing network that forms a high-resistance pathway. Bottom: calibrated IR frames at successive times after current onset; pronounced hotspots develop at and around the damaged region, confirming that the local defect — identified non-destructively from the image — controls where the hotspot forms.

A smooth spatial variation in the mesh topology produces a corresponding temperature gradient. Figure S4 shows a sample whose mesh density varies along its length; under excitation the temperature field develops a matching gradient, hottest where the network is least dense. Quantitatively, extracting the local fill factor (metal coverage) along the length and comparing it with the IR temperature profile gives a clear anti-correlation (r = −0.74): the hotter end is the end with the lower fill factor, because less metal coverage means a higher local sheet resistance and therefore greater Joule heating (P = I²R). This is consistent with the elevated temperatures observed in lower-density regions in the main text, and because the density distribution is recovered non-destructively from the image, the framework can anticipate where in-plane gradients and hotspots will develop prior to electrical testing.

Figure S4. A gradient in mesh topology produces a gradient in temperature. Top: micrograph showing the variation in mesh density along the strip. Bottom: calibrated IR frames at successive times after current onset; the temperature field develops a gradient along the sample, hottest at the lower-density end.

# Bibliography

1.	Sorel, S., Bellet, D. & Coleman, J. N. Relationship between material properties and transparent heater performance for both bulk-like and percolative nanostructured networks. *ACS Nano* vol. 8 4805–4814 (2014).

2.	Ji, S., He, W., Wang, K., Ran, Y. & Ye, C. Thermal response of transparent silver nanowire/PEDOT:PSS film heaters. *Small* **10**, 4951–4960 (2014).

3.	Matula, R. A. Electrical Resistivity of Copper, Gold, Palladium and Silver. *J. Phys. Chem.* **8**, 1147–1298 (1979).

4.	Cheng, Z., Liu, L., Xu, S., Lu, M. & Wang, X. Temperature dependence of electrical and thermal conduction in single silver nanowire. *Sci. Rep.* **5**, (2015).

5.	Furukawa, G., Saba, W. & Reilly, M. Critical Analysis of the Heat-Capacity Data of the Literature and Evaluation of Thermodynamic Properties of Copper, Silver, and Gold from 0 to 300K. (1968) doi:10.7312/schw92626-006.

6.	Goodfellow. Material Properties. (2022).

7.	Bowler, N. & Huang, Y. Electrical conductivity measurement of metal plates using broadband eddy-current and four-point methods. *Meas. Sci. Technol.* **16**, 2193–2200 (2005).

8.	Adineh, M. & Doostmohammadi, H. Microstructure, mechanical properties and machinability of Cu–Zn–Mg and Cu–Zn–Sb brass alloys. *Mater. Sci. Technol. (United Kingdom)* **35**, 1504–1514 (2019).

9.	Wilski, V. H. Die spezifische Wärme des Polyvinylbutyrals. *Die Angewandte Makromolekulare Chemie* vol. 6 101–108 (1969).
